# Survival Without Sequelae From Severe Acidosis Due to Sepsis and Acute Kidney Injury Following Colitis: A Case Report

**DOI:** 10.7759/cureus.84939

**Published:** 2025-05-28

**Authors:** Sanjoy George, Nishad Raveendran, Ateeq Omer

**Affiliations:** 1 Critical Care Medicine, Holy Family Hospital, Thodupuzha, IND; 2 Nephrology, Holy Family Hospital, Thodupuzha, IND; 3 Emergency Medicine, Holy Family Hospital, Thodupuzha, IND

**Keywords:** acute kindey injury, colitis, metabolic acidosis, severe acidosis, severe sepsis

## Abstract

When the pH is significantly low, metabolic acidosis is considered severe, and such a low level is typically regarded as incompatible with life. Despite being an indication of a bad prognosis, severe acidosis alone is not a strong enough predictor of outcome to support the refusal of resuscitation. Timely interventions can sometimes achieve recovery from extremely severe acidosis. Here, we report a case of severe metabolic acidosis with a pH less than 6.5 associated with sepsis and acute kidney injury, who survived without any functional deficits by timely resuscitation and a multidisciplinary approach.

## Introduction

Maintaining acid-base balance is essential for healthy physiology. Systemic acid-base balance is typically well-regulated, with arterial pH ranging from 7.36 to 7.44. Transmembrane transport, metabolic enzymes, and several vital cellular functions are all extremely sensitive to pH. Acute metabolic acidosis is common in seriously ill patients and, when severe, can be associated with a poor clinical outcome [1,2]. Acute metabolic acidosis is associated with increased morbidity and mortality because of its depressive effects on cardiovascular function, facilitation of cardiac arrhythmias, decreased cardiac output, arterial dilatation with hypotension, impaired oxygen delivery, decreased adenosine triphosphate (ATP) production, stimulation of inflammation, suppression of the immune response, and other adverse effects [3,4]. Metabolic acidosis is considered severe when the pH is less than 7.2, and a very low pH is usually considered incompatible with life [5]. Severe metabolic or mixed acidemia was associated with a mortality rate of 57% in the ICU [6].

## Case presentation

A 76-year-old woman presented to us with complaints of loose stools and vomiting for two days. She has a history of type 2 diabetes mellitus and systemic hypertension for the last eight years and was on insulin and the tablet amlodipine 5 mg twice daily. There was no history of any other significant illness in the past. Her complaints started following the consumption of pork meat two days prior to presentation. She had eight to 10 episodes of loose stools per day for the last two days. She was not on metformin, and there was no history suggestive of any toxin ingestion. On arrival at the hospital, her Glasgow Coma Scale was 4/15. Her pulse was 98 beats per minute, blood pressure was 60/40 mmHg, temperature was 98.6°F, and SpO_2_ was 52% on room air. Arterial blood gas analysis showed a pH of less than 6.5, bicarbonate was undetectable, lactate was 17.27 mmol/L, potassium was 7.6 mmol/L (Table 1), and serum creatinine was 6.4 mg/dL.

**Table 1:** Arterial blood gas analysis cnc*: *could not calculate; cHCO₃: calculated bicarbonate; BE(ecf): base excess (extracellular fluid); cSO₂: calculated O₂ saturation; Na⁺: sodium; K⁺: potassium; Ca^+^⁺: calcium; Cl⁻: chloride; cTCO₂: calculated total CO₂; AGapK: anion gap (potassium included); Hct: hematocrit; cHgb: hemoglobin (concentration); BUN: blood urea nitrogen

Test	Results	Reference range
PH	< 6.500	7.350 – 7.450
pCO_2_	25.8 mmHg	35.0 – 48.0 mmHg
pO_2_	169.2 mmHg	83.0 – 108.0 mmHg
cHCO_3_	cnc*	21.0 – 28.0 mmol/L
BE(ecf)	cnc*	-2.0 – 3.0 mmol/L
cSO_2_	cnc*	94.0 – 98.0 %
Na+	127 mmol/L	138 – 146 mmol/L
K+	7.6 mmol/L	3.5 – 4.5 mmol/L
Ca++	0.88 mmol/L	1.15 – 1.33 mmol/L
Cl-	105 mmol/L	98 – 107 mmol/L
cTCO_2_	cnc*	22.0 – 29.0 mmol/L
AGapK	cnc*	10 – 20 mmol/L
Hct	26	38 – 51 %
cHgb	8.9 g/dL	12.0 – 17.0 g/dL
BE(b)	cnc*	-2.0 – 3.0 mmol/L
Glu	182 mg/dL	74 – 100 mg/dL
Lactate	17.27 mmol/L	0.56 – 1.39 mmol/L
BUN	58 mg/dL	8 – 26 mg/dL
Crea	5.99 mg/dL	0.51 – 1.19 mg/dL
BUN/Crea	9.8 mg/mg	12.0 – 20.0 mg/dL

The patient had a triple acid-base disorder: high anion gap metabolic acidosis, respiratory acidosis, and metabolic alkalosis. Her serum albumin was 3.3 mg/dl, and lipase was 88. Her random blood glucose level was 180 mg/dl, and HbA1c was 8.8%. Hemogram showed neutrophilic leukocytosis (hemoglobin 8.5, total count 15300, platelet count 289000) and increased C-reactive protein (CRP) 54.25 and procalcitonin (3.66).

**Figure 1:**
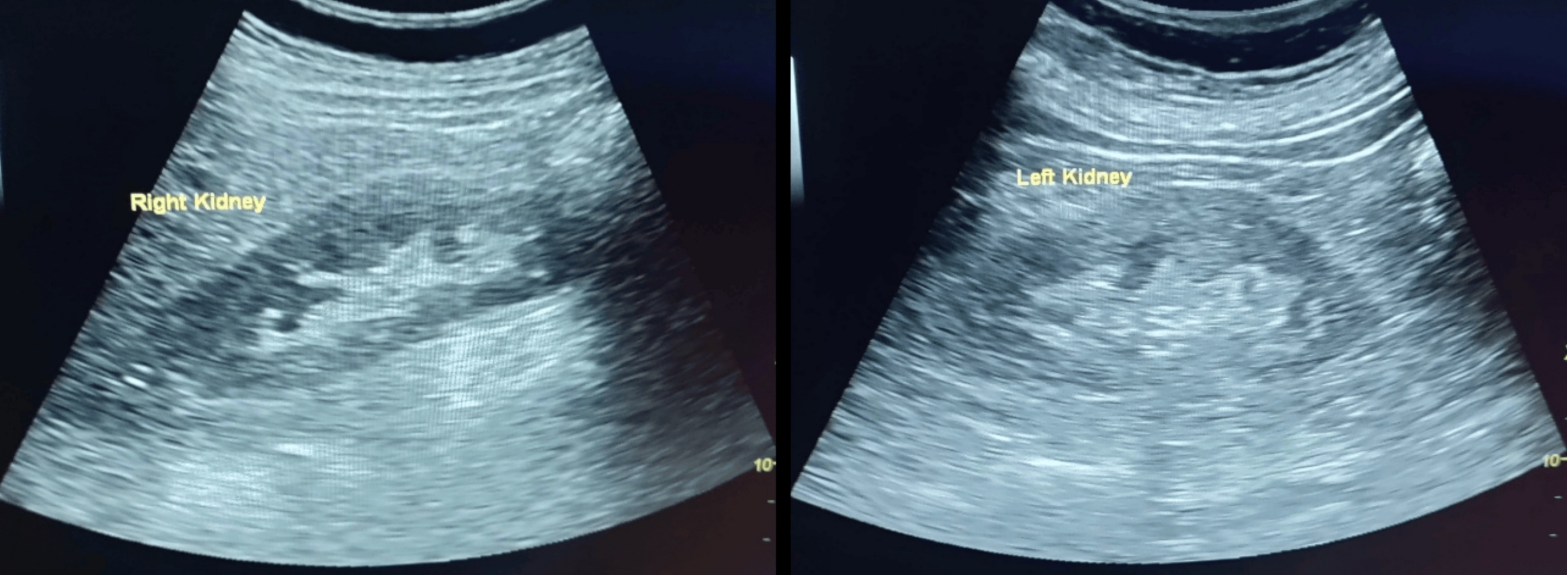
Ultrasound of the abdomen image showing both kidneys

She was intubated and started on mechanical ventilatory support. A bolus of intravenous (IV) fluid was given, and as blood pressure was not maintained, norepinephrine and vasopressin support were started. Intravenous bicarbonate was given, and she was started on broad-spectrum antibiotics after taking blood samples for culture and sensitivity. Her blood sugar levels were closely monitored, and insulin infusion was given. After six hours, she remained anuric, and a repeat arterial blood gas (ABG) showed mild improvement in pH (6.54), but lactate had increased to 21 mmol/L. Even though continuous renal replacement therapy (CRRT) is the preferred modality for treating patients in shock due to its superior hemodynamic stability and reduced risk of dialysis disequilibrium syndrome [7], she was taken for sustained low-efficiency dialysis (SLED) as the facility for CRRT was not available. Ultrasonography of the abdomen showed normal-sized kidneys with no evidence of obstruction (Figure 1) and diffuse wall thickening of the small and large intestines, suggestive of colitis.

Her condition improved with intravenous antibiotics and other supportive measures, and two sessions of SLED and ionotropic supports were tapered and stopped. She was weaned off ventilatory support and was extubated. Blood and stool cultures didn’t yield any growth, and lactate came down to 2.4.

She had a complete neurological recovery without any deficits, even though she didn’t remember the events. She had adequate urine output and didn’t require further renal replacement therapy. She was discharged home in a stable condition. On her follow-up visit, her serum creatinine had decreased to 3.3 mg/dl with good urine output. She was lost to follow-up after that.

## Discussion

Few studies in the literature have, as far as we are aware, documented a patient who survived a very low arterial blood pH due to metabolic acidosis. A case with an arterial blood pH of 6.48, which developed following status epilepticus and in which the patient recovered successfully, is reported [8]. A 62-year-old man who was taking metformin was reported to have hypothermia (core body temperature of 29ºC) and uncompensated respiratory acidosis and lactic acidosis coupled with an arterial blood pH of 6.38 [9]. Another case report is of a 24-year-old man who, following near-drowning cardiac arrest, developed severe metabolic acidosis with an arterial blood pH of 6.33 [10]. The lowest arterial blood pH reported in the literature (6.30) was described as occurring in an 84-year-old man and was related to metformin use, but more data are not available [11]. Our patient was an elderly female with significant comorbidities who presented with features of severe sepsis. The presence of hypotension, lactic acidosis, and renal failure is associated with poor outcomes in patients with sepsis. A very low pH is considered incompatible with life. However, timely and stepwise treatment, including renal replacement therapy, led to the patient's complete recovery. Even though survival without any deficit from a very low pH is rare, unbiased, timely, and protocol-based multidisciplinary resuscitation may often lead to favorable outcomes.

## Conclusions

Even simple gastroenteritis can progress to sepsis, multi-organ dysfunction, and death, especially in the elderly. Prompt protocol-based resuscitation is crucial for surviving sepsis. Survival without sequelae from very low pH may be rare, but not impossible. A very low pH should not be a reason to discontinue resuscitation. Decisions about resuscitation and treatment should be guided by the results of an overall clinical evaluation.
